# Annual Commencement Exercises in the Buffalo Medical College

**Published:** 1869-02

**Authors:** 


### Annual Commencement Exercises in the Buffalo Medical College. List of Graduates.

The Annual Commencement Exercises of the Buffalo Medical College took place on the evening of February 24th, at St. James Hall, in presence of a large and highly appreciative audience, Prof. J. P. White, M. D., presiding. The stage was occupied by the Faculty, Curators and members of the Council.

Prof. T. F. Rochester delivered the Address to the graduating class. Robert A. Patchin, member of the class, gave the Valedictory.

Hon. Millard Fillmore, Chancellor of the University, conferred the degree of Doctor in Medicine upon the following gentlemen:

| | |
|---|---|
| Edgar Leonard DeWitt, | Elmira, Chemung Co., N. Y. |
| John Mathew Dee, | Stamford, Welland Co., Ontario. |
| Leonard Crysler, | St. Davids, Lincoln Co., Ontario. |
| Frank Beaty Gallery, | Mount Read, Monroe Co., N. Y. |
| Joseph Ward Stone, | Niagara, Lincoln Co., Ontario. |
| John Joseph Burke, | Buffalo, Erie Co., N. Y. |
| Lysander S. White, | Trumbull's Corners, Tompkins Co., N. Y. |
| Robert Acker Patchin, | Livonia, Livingston Co., N. Y. |
| Frank Albert Jones, | Charlotte, Monroe Co., N. Y. |
| Erastus Gilbert Harding, | Bethany, Genesee Co., N. Y. |
| Seneca Allen, | Cuba, Allegany Co., N. Y. |
| Dillon Francis Acker, | Hannibal, Oswego Co., N. Y. |
| George Lewis Beach, | Dundas, Wentworth Co., Ontario. |
| John Thomas Brereton, | Buffalo, Erie Co., N. Y. |
| Wilber Harmon Greene, | Lyons, Wayne Co., N. Y. |
| Macy B. Searles, | Wales Centre, Erie Co., N. Y. |
| William Pardee, | Akron, Erie Co., N. Y. |
| John Hale Taylor, | Brockport, Monroe Co., N. Y. |
| Dwight Dickinson, | Jamestown, Chautauqua Co., N. Y. |
| Robert Lewis Kinkle, | New York City, N. Y. |
| William Henry Oviatt, | Utica, Dane Co., Wis. |
| William Nelson Greene, | Hopbottom, Susquehanna Co., Pa. |
| George Arthur Goodrich, | Cassadaga, Cattaraugus Co., N. Y. |
| Simon Augustus Freeman, | Canton, Norfolk Co., Mass. |
| Dwight Gustavus Hubbard, | Weathersfield, Wyoming Co., N. Y. |
| Wellington Mortimer Cheney, | Sandusky, Cattaraugus Co., N. Y. |
| William Richter Tupper, | Bay City, Bay Co., Mich. |
| Samuel Ezra Thayer, | Borodine, Onondaga Co., N. Y. |
| Julius Jerome Boyle, | Syracuse, Onondaga Co., N. Y. |
| George Wright Pattison, | Buffalo, Erie Co., N. Y. |

Albert De Alton Atwood, - - - Lockport, Niagara Co., N. Y.
Manhattan Pickett, - - - - Columbus, Warren Co., Pa.
Ransom Cary Sloan, - - - - - - Buffalo, Erie Co., N. Y.
James Monroe Andrews, - - - Rochester, Monroe Co., N. Y.

